# Continuous influenza virus production in a tubular bioreactor system provides stable titers and avoids the “von Magnus effect”

**DOI:** 10.1371/journal.pone.0224317

**Published:** 2019-11-05

**Authors:** Felipe Tapia, Daniel Wohlfarth, Volker Sandig, Ingo Jordan, Yvonne Genzel, Udo Reichl

**Affiliations:** 1 Max Planck Institute for Dynamics of Complex Technical Systems, Magdeburg, Germany; 2 ProBioGen AG, Berlin, Germany; 3 Chair for Bioprocess Engineering, Otto-von-Guericke-University Magdeburg, Magdeburg, Germany; Istituto Zooprofilattico Sperimentale delle Venezie, ITALY

## Abstract

Continuous cell culture-based influenza vaccine production could significantly reduce footprint and manufacturing costs compared to current batch processing. However, yields of influenza virus in continuous mode can be affected by oscillations in virus titers caused by periodic accumulation of defective interfering particles. The generation of such particles has also been observed previously in cascades of continuous stirred tank reactors (CSTRs) and is known as the “von Magnus effect”. To improve virus yields and to avoid these oscillations, we have developed a novel continuous tubular bioreactor system for influenza A virus production. It was built using a 500 mL CSTR for cell growth linked to a 105 m long tubular plug-flow bioreactor (PFBR). Virus propagation took place only in the PFBR with a nominal residence time of 20 h and a production capacity of 0.2 mL/min. The bioreactor was first tested with suspension MDCK cells at different multiplicities of infection (MOI), and then with suspension avian AGE1.CR.pIX cells at a fixed nominal MOI of 0.02. Maximum hemagglutinin (HA) titers of 2.4 and 1.6 log_10_(HA units/100 μL) for suspension MDCK cells and AGE1.CR.pIX cells, respectively, were obtained. Flow cytometric analysis demonstrated that 100% infected cells with batch-like HA titers can be obtained at a MOI of at least 0.1. Stable HA and TCID_50_ titers over 18 days of production were confirmed using the AGE1.CR.pIX cell line, and PCR analysis demonstrated stable production of full-length genome. The contamination level of segments with deletions (potentially defective interfering particles), already present in the virus seed, was low and did not increase. Control experiments using batch and semi-continuous cultures confirmed these findings. A comparison showed that influenza virus production can be achieved with the tubular bioreactor system in about half the time with a space-time-yield up to two times higher than for typical batch cultures. In summary, a novel continuous tubular bioreactor system for cell culture-based influenza virus production was developed. One main advantage, an essentially single-passage amplification of viruses, should enable efficient production of vaccines as well as vectors for gene and cancer therapy.

## Introduction

Influenza viruses are a major threat for human and animal health. Influenza viruses have an approximate size of 100 nm and are characterized by an enveloped structure with a negative-sense RNA genome. The genome is divided in 7–8 separated segments coding for more than 10 proteins depending on strains [1]. Hemagglutinin (HA) and neuraminidase (NA), the two main viral glycoprotein antigens, are located in the virus membrane. Infectious units are transmitted via air droplets and cause sudden fever and severe morbidity, sometimes leading to the death of the patients either directly or via bacterial sequelae. The most effective approach to control the disease is by vaccination [2]. Although influenza vaccine production capacity increased to 6.4 billion doses in 2015, providing enough vaccines remains challenging especially in a pandemic situation [3].

The main technology platform for influenza virus production is based on the infection and harvest of embryonated-chicken eggs. Despite the annual need for millions of eggs and dependence on a complex logistic, this technology is still considered efficient for production of seasonal influenza vaccines [4]. However, limitations regarding response time and scalability in case of a pandemic is a main public concern [3]. To alleviate these limitations, animal cell culture and bioreactor technology has been introduced for influenza vaccine production in Europe and the United States in the past two decades [5]. Typically, cells are grown to high concentrations (2–6×10^6^ cells/mL) and, once the desired cell concentration is reached, the culture is infected and harvested after about 2–3 days. More recently, a recombinant influenza vaccine using the baculovirus expression system has also been approved for commercialization [5]. Despite small differences in process operation and parameters among these platforms, all processes are basically operated in batch mode. Moving from batch to continuous production could significantly improve volumetric productivity (virus produced/[(time)×(volume of cultured media used)]) and reduce the manufacturing footprint [6]. Continuous production is currently not only promoted by various manufacturers of recombinant CHO cell-based biologicals, but also by regulatory agencies [7].

Cascades of stirred tank bioreactors have been used since the 1960’s for production of viruses in continuous mode [8]. This included adenovirus, poliovirus, baculovirus, picornavirus [9], influenza virus [10], and Modified Vaccinia Ankara virus [6]. The cascades are characterized by one continuous stirred tank reactor (CSTR) for cell growth and at least one CSTR in series for virus infection and propagation. The use of a cascade of CSTRs is a good option for production of genetically stable viruses such as MVA, but suffers from low productivity levels when less stable viruses such as influenza virus and baculovirus are propagated [10] [11]. In particular, these viruses show oscillations in virus concentration over cultivation time that can be explained by the accumulation of defective interfering particles (DIPs) in the virus population. DIPs have deletions in genes required for replication so that they depend on co-infections with standard viruses (STV) with full-length (FL) genome for successful propagation [12]. At high DIP concentrations, the replication of the STV is reduced, and the infectious units are washed out of the CSTR. This generates oscillations in the virus titers, which is known as “von Magnus effect”, in honor to Preben von Magnus, who discovered these “incomplete forms of influenza virus” in 1950’s [13].

An alternative continuous production system could be a tubular plug-flow bioreactor (PFBR) [14]. Tubular bioreactors have been used, for example, for waste water treatment [15], for production of bioethanol [16], and for production of algae [17]. Tubular bioreactors have unique properties such as a reduced back-mixing and a large surface-to-volume ratio that are useful for a variety of biotechnological applications. In contrast to CSTRs, reagents and products do not accumulate in the tubular bioreactor volume over process time [18]. The reduced back-mixing within the PFBR volume and the possible compartmentalization of the fluid via air bubbles ensure that DIPs produced within a compartment do not interact with recently infected cells at neighboring compartments. This combination can in theory reduce co-infection of cells compared to a normal batch or two-stage CSTR infection, and therefore significantly minimize the amount of DIPs produced. Therefore, if enough time for virus replication inside the PFBR is provided, the von Magnus effect is avoided, and stable titers can be expected in the harvest.

In this work, a continuous tubular bioreactor system for influenza A/PR/8/34 (H1N1) virus production is presented. The system consisted of a CSTR operated in chemostat mode for cell growth linked to a PFBR for virus propagation. Units operations such as point of infection (POI), virus stock (VS), air injection and medium stock are introduced. Nominal values of residence time (RT) and multiplicities of infection (MOI) were set for each cultivation. Because flow rates and cell concentrations were adjusted during some experiments, the actual value of RT and MOI are also reported. First, results from cultivations with suspension MDCK cells infected at different MOI are shown to complement previous findings [19] and to discuss options for process optimization. In addition, results for influenza virus production at defined MOIs in an avian suspension cell (AGE1.CR.pIX) are presented. The results obtained demonstrate that DIP-induced oscillations in virus titers, which have been observed previously using avian suspension cells in cascades of CSTRs [10], can be avoided with the described system.

## Materials and methods

### Cell lines and influenza virus strain

Suspension MDCK.SUS2 cells (collaboration with Prof. Klaus Scharfenberg, University of Applied Sciences Emden-Leer, Germany) were used and inoculated at 0.5×10^6^ cells/mL into the CSTR. Cells were grown in the chemically defined medium Smif8 (also obtained from Prof. K. Scharfenberg) and supplemented with 4 mM L-glutamine and 4 mM pyruvate immediately before the cultivation. MDCK.SUS cells with passage number 40 and 58 were used for the experiments described in sections 3.1 and 3.2.

The avian suspension cell line AGE1.CR.pIX (ProBioGen AG, Germany) was grown in a chemically defined medium CD-U3 (Biochrom-Merck, Germany) supplemented with 2 mM of L-glutamine (Sigma-Aldrich, Germany), 2 mM L-alanine (Fluka Analytical, Sigma-Aldrich, Germany) and 10 ng/mL Long^®^R^3^IGF-I (SAFC Biosciences, USA). AGE1.CR.pIX cells were inoculated at a concentration of 0.8×10^6^ cells/mL, and passaged in shaker flasks at 37°C, 5% CO_2_ in air, and 185 rpm.

The virus strain used to infect MDCK.SUS2 cells was influenza A/PR/8/34 (RKI) virus adapted to adherent MDCK cells (1.28×10^8^ TCID_50_/mL). For infection of AGE1.CR.pIX cells, this virus was adapted over four passages to propagation in AGE1.CR.pIX cells (1.48×10^7^ TCID_50_/mL) as reported previously [10].

### Continuous tubular bioreactor system: Set-up and operation

**Fig 1:**
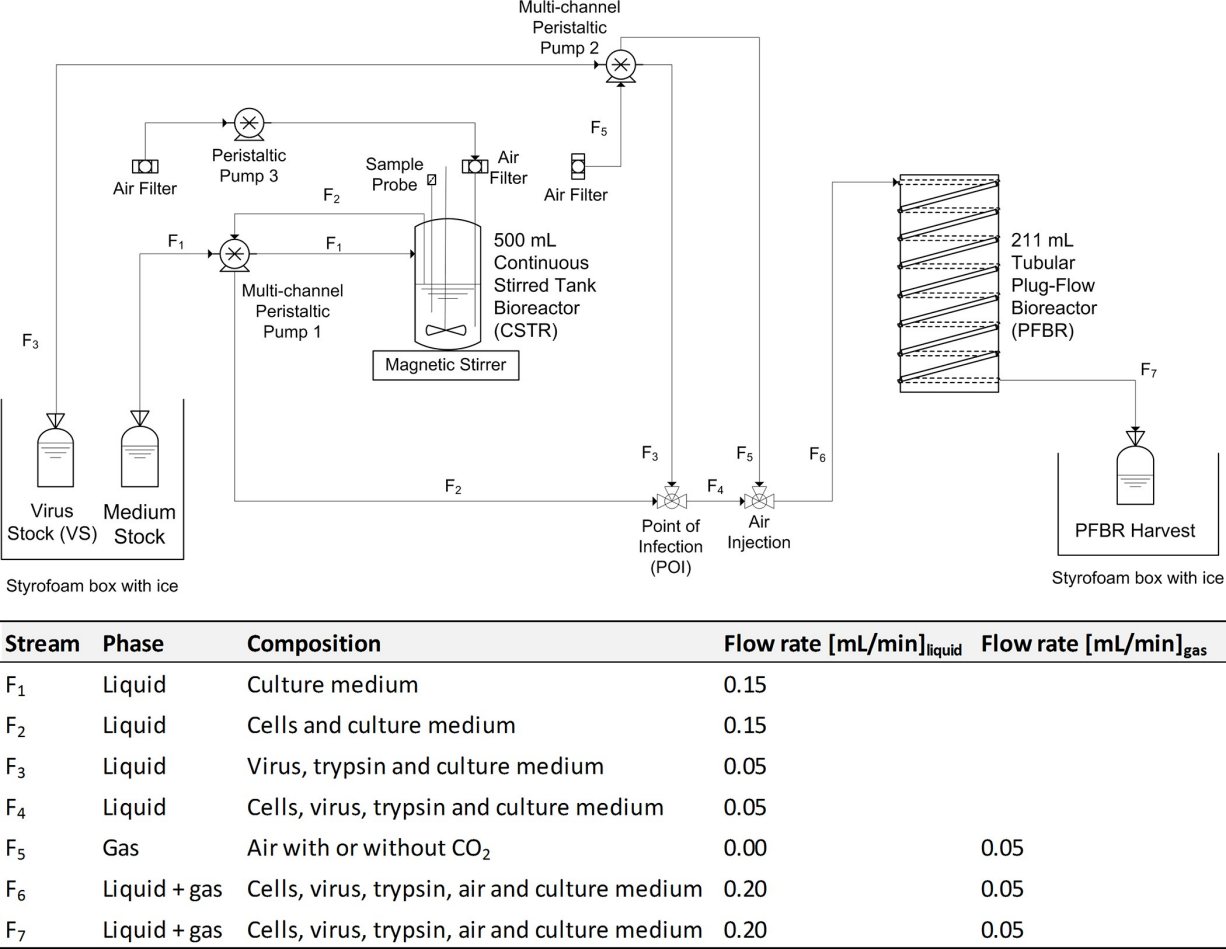
Process flow diagram of the continuous tubular bioreactor system for influenza virus production. The system was built with a CSTR and a coiled tubular plug-flow bioreactor (PFBR) in series. The complete bioreactor system was installed inside a cultivation room at 37°C. The CSTR was operated as a chemostat with a dilution rate of approx. 0.9×μ_max_. The PFBR was constructed using a transparent silicone tube that was coiled around a PLEXIGLAS^®^ XT tube of 20 cm internal diameter and 1 m height.

A continuous tubular bioreactor system consisting of a 500 mL CSTR followed by a 211 mL PFBR with a nominal production capacity of 12 mL/h was established as shown in Fig 1, and the system was operated in a cultivation room at a controlled and stable temperature of 37°C. The first bioreactor unit (CSTR; pitch-blade stirrer; Dasgip, Germany) was inoculated with either MDCK or AGE1.CR.pIX cells and operated at 37°C, 150 rpm, pH 7.0–7.3, and 350 mL working volume (WV). Aeration of the CSTR was provided with a tube connected to two 0.2 μm air filters (Sartorius, Switzerland) that injected bubbles into the CSTR. The feed medium (Smif8 or CD-U3) bottle was stored in a styrofoam box with ice (which was replaced twice a day).

The start-up phase was initiated by priming the system with phosphate-buffered saline (PBS) for up to 72 h to check for leakage and to control correct operation of pumps. Once the system reached a stable flow pattern, the PBS was removed, and the CSTR was seeded with cells and fresh medium. The continuous cultivation was initiated either immediately after cell seeding or after some hours, but only PBS was pumped initially from the VS bottle to prime the PFBR. To start the infection, the PBS in the VS bottle was replaced by fresh medium containing 42.8 trypsin units/mL (Gibco, UK) and between 10^4^−10^5^ TCID_50_/mL (based on desired MOI). 15 min after the virus was added to the stock bottle, the infection was initiated (this corresponds to the time it takes for the transfer of the virus solution from the stock bottle to the POI). First drops of virus-containing culture medium can be collected at the tube outlet about 20 h after the VS addition (corresponding to the RT of the tubular bioreactor, Eq 2).

The MOI at the POI was determined with the following equation:

$$MOI=\frac{F_{3}\times {\left[virus\;concentration\right]}_{Virus\;Stock}}{F_{2}\times {\left[cell\;concentration\right]}_{CSTR}}$$ (1)

with the flow rates of the VS (F_3_) and the CSTR (F_2_), respectively. To avoid virus degradation, the stock solution was replaced every 48 h, and the VS bottle was stored together with the medium stock bottle in a styrofoam box with ice. The flow rates used are described in Fig 1. Samples of 3–5 mL were taken once a day from the CSTR, and twice a day from the harvest bottle for measuring cell concentration, viability, off-line pH value, metabolite (glucose and lactate) and virus concentrations (HA and TCID_50_ titers).

The average RT in the tubular bioreactor was determined with the following equation:

$$RT=\frac{L}{V_{T}}$$ (2)

where the RT (h) is a function of the length (L) of the PFBR and the linear velocity inside the tube (V_T_), respectively. The latter was calculated based on the flow at the tube outlet (mL/min) divided by the transversal area of the tube (cm^2^). The flow rate at the tube outlet was determined by measuring the collected PFBR harvest volume twice a day. The flow diagram of Fig 1 can be read as follows. Cells are first grown in the CSTR in batch mode to a final concentration depending on cell line. Then, continuous cultivation is started and cells are produced in the CSTR with addition of fresh medium from the medium stock bottle (F_1_, Fig 1), and transferred by a peristaltic pump to the POI (F_2_), where infection takes place. The influenza virus seed is stored at 0°C in the VS bottle (F_3_) and replaced every 48 h using fresh trypsin, viruses and medium. Air is 0.2 μm filtered and injected into the system to provide oxygen to the cells (F_5_). The mixture of cells, virus and air bubbles (F_6_) travels through the PFBR with a nominal RT of 20 h. Finally, the product (F_7_) is collected in a harvest bottle and sampled twice a day for analysis.

Finally, the Reynolds number (Re) in the tubular bioreactor was calculated with the following equation:

$$Re=\frac{\rho \times V_{T}\times D}{\upsilon }$$ (3)

where *ρ* is the density of the fluid (993 kg/m^3^, water at 37°C), V_T_ is the mean velocity of the fluid (m/s), D is the hydraulic diameter of the tube (m) and *υ* is the dynamic viscosity of the fluid (0.000691 kg/(m×s), water at 37°C).

### Continuous influenza virus production in MDCK.SUS2 cells in the tubular bioreactor system

To test the feasibility of influenza virus production in the tubular bioreactor system, the first experiment was performed using the suspension MDCK.SUS2 cell line. Cells were seeded at 1×10^6^ cells/mL in the CSTR and the continuous culture was immediately initiated with a dilution rate (D) of 0.9×μ_max_ (D = F_2_/WV_CSTR_, Fig 1) _._ The PFBR was fed with cell culture from the CSTR and virus from the VS immediately after starting the continuous mode. The experiment was maintained for 23 days. In addition to the cell concentration, HA and TCID_50_ titers were determined. The VS used in this experiment had an infectious titer of 7×10^4^ TCID_50_/mL. This resulted in a nominal MOI of 0.025 at the POI. The nominal RT in the tubular reactor was 20 h.

### Impact of MOI on virus production in suspension MDCK cells

To test the impact of different MOI on virus titers of the PFBR harvest, one additional experiment of 240 h (10 days) with MDCK.SUS2 cells was carried out. The experiment was started using a CSTR that was already running with MDCK cells at 1.2×10^6^ cells/mL and with a dilution rate of 0.9×μ_max_. More MDCK cells, taken from a shake flask, were added to the CSTR to reach a concentration of 5×10^6^ cells/mL, similar to the cell concentration observed in a previous successful experiment [19]. The RT of the PFBR was adjusted to 18 h by increasing the rotational speed of Pump 2. The MOI was changed at different time points of cultivation by increasing the VS concentration in four phases from 0.2×10^6^ TCID_50_/mL (phase I), to 0.7×10^6^ TCID_50_/mL (phase II), to 6.2×10^6^ TCID_50_/mL (phase III), and back to 0.2×10^6^ TCID_50_/mL (phase IV). Samples from the PFBR harvest for HA and flow cytometry analysis were taken in all phases. A control experiment to cover the whole MOI range was performed infecting MDCK.SUS2 cells in shake flasks with MOI similar to phase I, II and III.

### Continuous influenza virus production in AGE1.CR.pIX cells in the tubular bioreactor system

Frensing et al. [10] showed that continuous influenza A virus production with the avian cell line AGE1.CR in cascades of CSTRs leads to oscillations in the virus titers known as “von Magnus effect”. To challenge these results, the avian cell line AGE1.CR.pIX was used for continuous influenza A virus production in the continuous tubular bioreactor system. The tubular bioreactor was operated as indicated in section 2.2 and continuous virus production was maintained over three weeks. In addition, control experiments using batch and semi-continuous cultures were carried out and compared to the tubular bioreactor system (see below). Cell concentration, cell viability, HA and TCID_50_ titers, and segment-specific PCR for identification of defective segments were determined from 4–5 mL samples. Two selected cultivation runs named tubular cultivation A and B are here presented. Tubular cultivation A was started from a newly established bioreactor setup. Tubular cultivation B was started after a different experiment was finished by flushing out the complete bioreactor with PBS, and then seeding fresh cells in the CSTR and preparing a new virus stock.

### Control experiments in AGE1.CR.pIX cells: Batch and semi-continuous cultures

For batch control experiments, a volume of 30 mL and 50 mL of AGE1.CR.pIX cells were taken from the CSTR of the tubular bioreactor system and infected in batch mode in shaker flasks (150 mL shakers with baffles, Corning, USA). With cell concentrations of about 5–6×10^6^ cells/mL, a 100:58 dilution (culture volume: fresh CD-U3 medium) was performed with VS, identically prepared as in the PFBR experiments, to mimic the infection conditions at the POI in the PFBR. The infection was carried out with an MOI of 0.025 using the influenza virus strain A/PR/8/34 in fresh medium before adjusting to the final 100:58 dilution. HA and TCID_50_ titers, and segment-specific PCR were analyzed.

A semi-continuous two-stage cultivation system (SSC) using two shaker flasks in series was used for reproducing the results of the two-stage continuous cultivations described previously [10]. The SSC system had a nominal production capacity of 3 mL/h and was operated for two weeks. It consisted of a 120 mL WV shaker flask for cell production (Small Cell Bioreactor, SCB; without baffles, 5% CO_2_ and 185 rpm; Corning, USA), and a 65 mL WV shaker flask for influenza virus propagation (Small Virus Bioreactor, SVB). An MOI of 0.025 was used. The methodology for sampling, medium exchanges and harvesting, as well as the equations required for operating this semi-continuous cultivation system is described in detail in [6]. HA titers, TCID_50_ titers, and segment-specific PCR were analyzed.

### Sampling and analytics

#### Cell concentration and viability

Concentration and viability of AGE1.CR.pIX and MDCK.SUS2 cells were determined using a *ViCell*^*TM*^ *XR cell viability analyzer (Beckman Coulter GmbH*, *Germany)* with a standard deviation of 5% for concentration measurements [20]. Samples containing MDCK.SUS2 cells were treated with trypsin to reduce aggregation before cell counting as described previously [21].

#### Influenza virus titers

The total number of viral particles was determined from viral HA titration as described previously and is expressed in units of log_10_ (HA units/100 μL). [22]. The titer of infectious virus particles was measured using a TCID_50_ assay and is expressed in units of [TCID_50_/mL] [23]. In addition, PCR was performed for influenza A virus genome segments 1, 2, and 3 as described previously [10].

#### Flow cytometric analysis

The percentage of infected MDCK.SUS2 cells at the outlet of the tubular bioreactor was measured using flow cytometry (ImageStream®, Merck). A total of 0.5–1.0×10^6^ cells were fixed with a final concentration of 2% paraformaldehyde. Cells were trypsinized for 10 min after fixing. For viral nuclear protein (NP)-staining, a protocol using two antibodies was used [24]; the wavelengths for FITC excitation/emission were 488 and 642 nm, respectively.

#### Productivity indicators of the cultivation systems

The productivity of the tubular system can be described with the two parameters time yield (TY) and space-time yield (STY):

$${TY}_{t_{n}}=\frac{\sum_{0}^{t_{n}}\left({Virions}_{{H,t}_{n}}\times V_{{H,t}_{n}}\right)}{t_{n}}$$ (4)

$${STY}_{t_{n}}=\frac{\sum_{0}^{t_{n}}\left({Virions}_{{H,t}_{n}}\times V_{{H,t}_{n}}\right)}{\sum_{0}^{t_{n}}\left(V_{{H,t}_{n}}\right)\times t_{n}}$$ (5)

**Fig 2:**
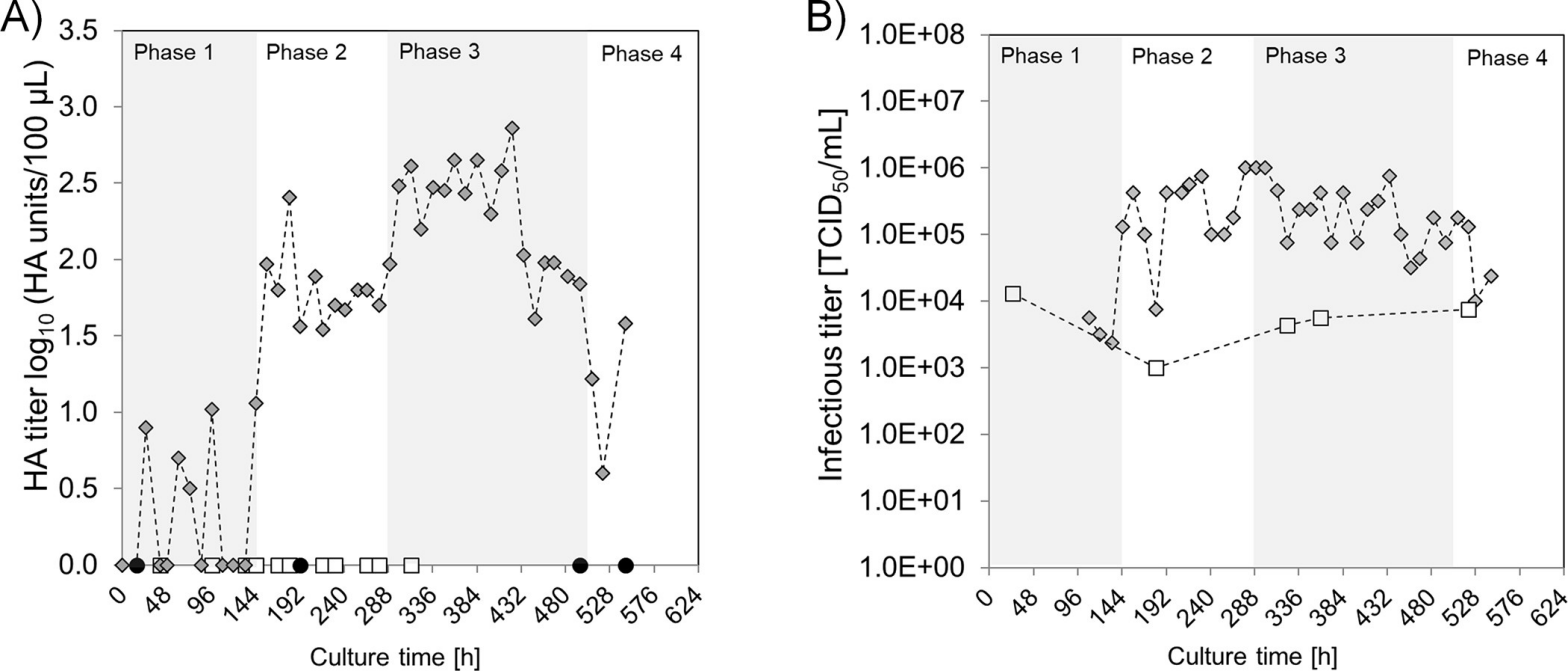
Influenza virus production using suspension MDCK.SUS2 cells (nominal MOI of 0.025, nominal residence time in the PFBR of 20 h) with the continuous tubular bioreactor system. A) HA titers of the PFBR harvest (closed diamonds), in the VS (open squares) and CSTR (closed circles) [19]. B) TCID_50_ titers of the PFBR harvest (closed diamonds) and VS (open squares).

Where t_n_ is the total operational time, ${Virions}_{{H,t}_{n}}$ is the concentration of virions (estimated from HA titer assuming that 1 virus particle binds to 1 cell) of a harvest at time t_n_ (if no harvest is taken at time t_n_ its value is zero), and $V_{{H,t}_{n}}$ is the harvest volume collected at time t_n_. Here, tubular and batch modes are compared with the assumption that the HA titer of the PFBR harvest is stable at 2.5 log_10_ (HA units/100 μL). This value is based on the best result obtained with suspension MDCK cells (Fig 2A, 384–432 h) and corresponds to 6.4×10^9^ virions/mL if 1 virion binds to 1 cell. Eqs 4 and 5 were also used for the estimation of the productivity of two hypothetical batch processes in single-use or stainless steel bioreactors with the same 711 mL WV as the tubular bioreactor system. The (maximum) productivity of these hypothetical batch cultivations was estimated using the same HA titer of 2.5 log_10_ (HA units/100 μL). Time zero (t_0_) of the tubular bioreactor system was the time in which the first virus-containing PFBR harvest drop was observed (approx. 20 h after the start of the infection at the “point of infection”). Time zero of the batch processes was the time of the first virus harvest.

The total number of virus particles produced over time was derived by multiplication of the STY with the accumulated volume and culture time (corresponding to the numerator of Eq 5).

## Results and discussion

### Continuous influenza A virus production in suspension MDCK.SUS2 cells in the tubular bioreactor system

The first functional cultivation with the tubular bioreactor system was operated for a total of 552 h (23 days). A stable cell concentration of 5–6×10^6^ cells/mL in the CSTR was achieved after one week of culture. The nominal RT of the PFBR was set to 20 h, however, the actual RT, determined by measuring the volume of harvest collected twice a day, was 21 h. Results for HA and TCID_50_ titers are depicted in Fig 2A and 2B, respectively. During the first 144 h of culture (phase 1), HA titers in the tubular bioreactor harvest were below the detection limit, and the TCID_50_ titers corresponded to the VS. This indicated that the virus did not replicate. Once the right conditions for virus propagation were reached, i.e. cell concentration, mixing, and pH (phase 2), an average HA titer of 1.6 log_10_(HA units/100 μL) and an average of 4×10^5^ TCID_50_/mL were obtained from harvest bottle samples, which indicated successful virus replication. Bioreactor system operation was stable until 288 h of culture, when MDCK.SUS2 cells started to grow in agglomerates resulting in cell sedimentation and clogging at some points of the tube. Accordingly, in phase 3, the flow rates (F_3_, Fig 1) were increased to washout the cell clumps. This led to a change in the actual RT from 21 to 18 h in the PFBR. To keep the nominal MOI of 0.025, the VS concentration was increased to 2×10^5^ TCID_50_/mL. For this phase, HA titers of approximately 2.5 log_10_(HA units/100 μL) and infectious virus titers up to 1×10^6^ TCID_50_/mL were obtained. From 504 h of culture (phase 4), the pH value of the manually-operated CSTR bioreactor decreased from 7.2 to 6.6 (due to overnight failure of the aeration pump). This resulted in a drop in the HA titers of the system to about 0.5 log_10_(HA units/100 μL), and the experiment was stopped.

### Impact of MOI on virus production in suspension MDCK cells

**Fig 3:**
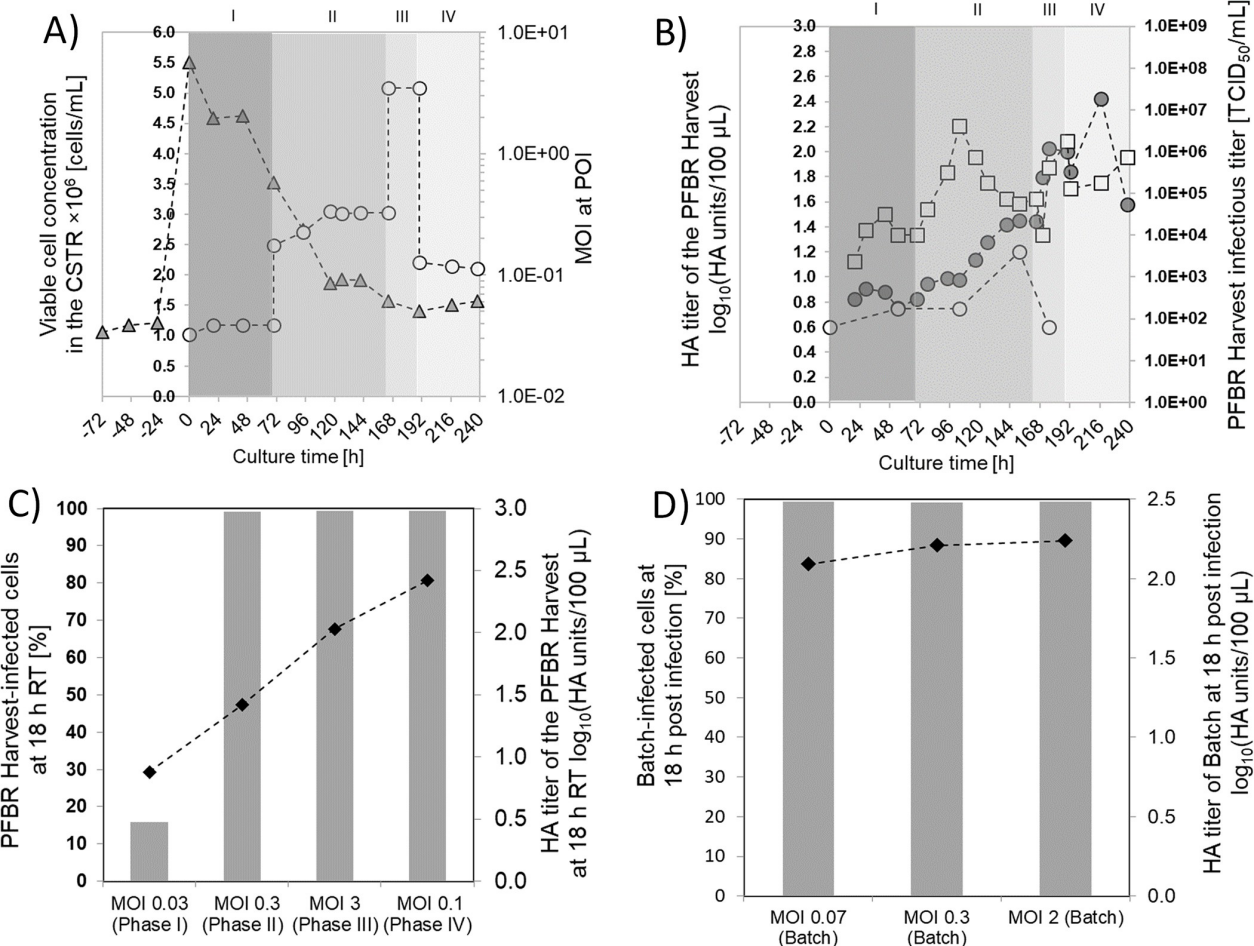
Impact of MOI regarding progress of infection and influenza A virus titers using MDCK.SUS2 cells in a continuous tubular bioreactor system. The PFBR had a residence time of 18 h. A) Viable cells (triangles) in the CSTR and actual MOI at the POI (circles). The different background colors show the four MOI phases. The MOI was modified by changing the concentration in the VS from 0.2×10^6^ TCID_50_/mL (Phase I) to 0.7×10^6^ TCID_50_/mL (Phase II), 6.2×10^6^ TCID_50_/mL (Phase III) and back to 0.2×10^6^ TCID_50_/mL (Phase IV). The MOI was calculated using Eq 1. B) HA titer in the PFBR Harvest (closed circles) and in the VS (open circles); TCID_50_ titers in the PFBR harvest (open squares). C) Percentage of infected cells in the tubular bioreactor harvest at four different MOI (grey columns) and maximum HA titer in the PFBR harvest for each MOI condition (closed diamonds). D) Control experiment of influenza A virus propagation at 18 h post infection in batch mode (shaker flask). The percentage of infected cells (grey columns) at three different MOI, and the HA titers (closed diamonds) at 18 h post infection are shown. Suspension MDCK.SUS2 cells were used in the continuous tubular bioreactor system and for the control experiment. Negative times in Fig A and B indicate that the CSTR was already running with MDCK.SUS2 cells and with PBS in the VS.

To investigate the impact of MOI on the virus titers of the PFBR harvest, different MOI were adjusted over the cultivation time. This was done by changing the VS concentration resulting in four MOI phases, as depicted in Fig 3 A (I, II, III and IV). A running CSTR with 1.2×10^6^ MDCK.SUS2 cells/mL was fed with more cells to obtain a concentration of 5.5×10^6^ cells/mL (0 h of culture, Fig 3A), similar to the steady-state concentration of the experiment described in 3.1. The cell concentration decreased the first 120 h of culture, possibly because the cells were of a different passage number, and a stable concentration of about 1.7×10^6^ cells/mL was reached only after 120 h. However, the main goal of this experiment, comparison of different MOI values, was not affected by this decrease in cell concentration. The MOI shown in Fig 3A was calculated for the cell concentration, flow rates and virus concentrations at the respective time points using Eq 1. The resulting MOI of phase I, II, III and IV were 0.03, 0.1, 3 and 0.1, respectively.

In phase I (MOI = 0.03, 0–70 h), the HA titer in the harvest (closed circles) was similar to the HA titer in the VS (open circles) with values around 0.9 log_10_(HA units/100 μL) (Fig 3B). In phase II (MOI = 0.3, 70–150 h), the HA increased up to 1.4 log_10_(HA units/100 μL) in the harvest, while the HA titer in the VS was near 0.7 log_10_(HA units/100 μL). Phase III (MOI = 3, 150–180 h) resulted in HA titers of 2.0 log_10_(HA units/100 μL), while the VS had titers near 1.2 log_10_(HA units/100 μL). Later, in phase IV (MOI = 0.1, 180–240 h), an HA titer as high as 2.4 log_10_(HA units/100 μL) was obtained (216 h) before dropping to 1.6 log_10_(HA units/100 μL).

The results obtained with these MOI variations showed that the HA titers obtained in the PFBR harvest increase with increasing MOI at the POI. Despite having been seeded at 5.5×10^6^ cells/mL, the concentration of MDCK.SUS2 cells in the CSTR after 120 h of culture was close to 1.7×10^6^ cells/mL, which was at least 2-fold lower compared to the MDCK.SUS2 concentrations obtained in previous batch cultivations [21] [25]. While a maximum HA titer of 2.4 log_10_(HA units/100 μL) was obtained at the outlet of the PFBR at 216 h of culture, previous batch experiments have shown HA titers up to 2.9 log_10_(HA units/100 μL) for cell concentrations in the range of 1.9–2.3×10^6^ cells/mL in STR and wave bioreactors [21]. However, increasing the viral titers of the continuous tubular bioreactor system is feasible with further optimization. A more specific comparison between a batch and continuous tubular bioreactor was made for AGE1.CR.pIX cells and will be presented in the following chapters. Finally, experiments with suspension MDCK cells showed that cell sedimentation in the tube can be an issue when cell agglomerates are present. Nevertheless, using high flow rates, tubular bioreactors can be operated over weeks without interruption.

For the lower MOI condition (0.03) obtained in phase I, only 16% of the cells collected at the PFBR harvest were infected (Fig 3C). In contrast, in phases II, III and IV (MOI of 0.3, 3, and 0.1, respectively) the percentage of infected cells was almost 100%. While phase I (lowest MOI condition) resulted only in an HA titer of 0.9 log_10_(HA units/100 μL) in the harvest, phase II, III and IV led to 1.5, 2.0 and 2.4 log_10_(HA units/100 μL), respectively. The HA titers obtained for high MOI conditions were similar to titers obtained in batch culture (Fig 3D). Control experiments indicated that a batch culture at 18 h post infection has about 100% of infected cells and an average HA titer of 2.2 log_10_(HA units/100 μL). Interestingly, despite the fact that 100% infected cells were found in the PFBR Harvest, a MOI of 3 resulted in a lower HA titer. Phase IV resulted in a HA peak titer of 2.4 log_10_(HA units/100 μL) at 216 h, however, the subsequent drop to 1.6 log_10_(HA units/100 μL) suggests that the peak might have been caused by cells infected in phase III that remained inside the tube (possible due to flow perturbation when changing the VS from phase III to IV). This result suggests that progress of infection under this condition is slower than in batch cultivations and a longer tube might be required to achieve higher titers. Hence, while operation of the tubular reactor under the actual laminar regime (Reynolds number about 30–60) allows to infect all cells at a MOI near 0.1, the RT should be increased to allow for an extension of virus spread and intracellular virus propagation to achieve batch-like titers. To use MOI below 0.1, the mixing conditions inside the tubular bioreactor have to be improved, e.g., by introducing static-mixers or mechanical mixing.

### Production of influenza virus in AGE1.CR.pIX cells in batch and semi-continuous reference experiments

**Fig 4:**
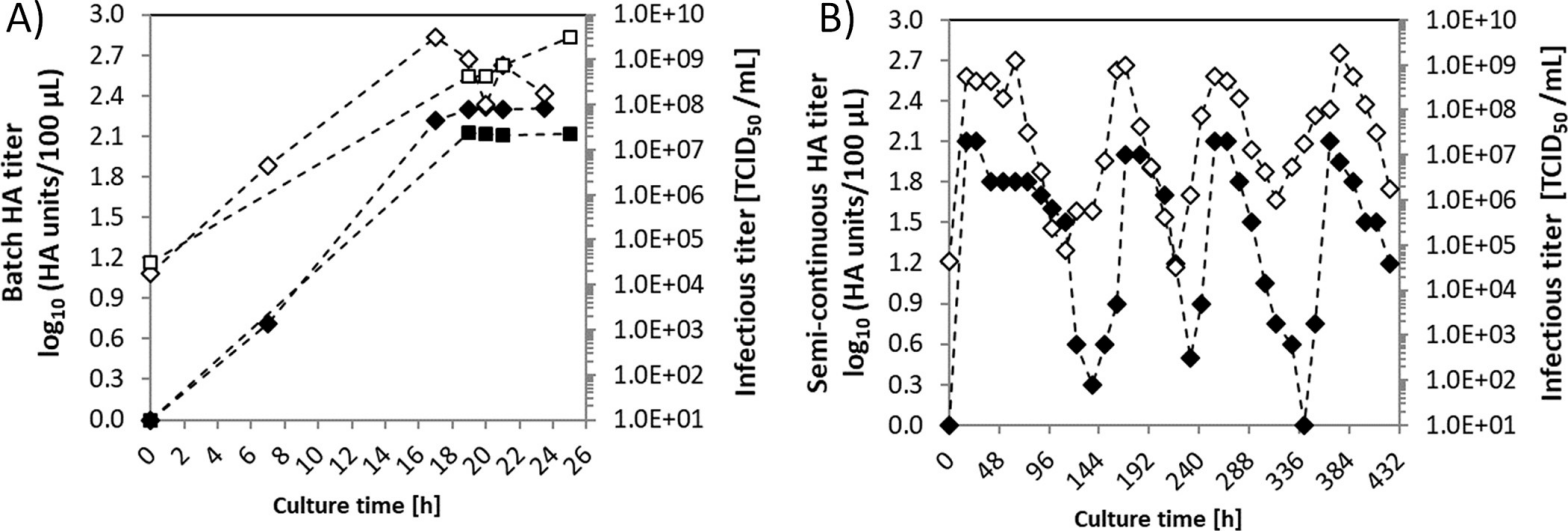
Influenza A/PR/8/34 virus propagation in AGE1.CR.pIX cells in batch and semi-continuous cultures. A) HA titers (closed symbols) and TCID_50_ titers (open symbols) of two batch cultures (diamond and square symbols). Batch cultures were initiated with cells of the CSTR of the continuous tubular bioreactor system (see Fig 1) after termination of continuous cultivations. B) Influenza HA titers (closed diamonds) and TCID_50_ titers (open symbols) of a semi-continuous culture performed in shake flasks as described previously [6].

To determine the maximum virus titers under perfect mixing conditions, and to confirm the presence of “von Magnus effect” in serial passage, influenza virus was propagated in AGE1.CR.pIX cells in batch and semi-continuous mode. Batch cultures were infected with an MOI of 0.025. Maximum infectious virus titers were observed 18 h post infection with a value of 1.0×10^9^ TCID_50_/mL. HA titers started to increase at 7 h post infection and maximum values of approximately 2.1 log_10_ (HA units/100 μL) were obtained at 20 h post infection (Fig 4A). Such high TCID_50_ titers with rather low HA values seem typical for AGE1.CR.pIX cells [26].

Influenza virus was also propagated in semi-continuous mode using the same MOI (Fig 4B). Virus levels oscillated over 450 h of culture. As in batch cultures, maximum HA titers of 2.1 log_10_ (HA units/100 μL) were observed at 24 h post infection, followed by a drop in the HA titers to values below the detection limit of the assay. Similarly, infectious virus titers oscillated between 1.0×10^9^ TCID_50_/mL in the upper range, and 1.0×10^5^ TCID_50_/mL in the lower range. Overall, the titers observed did not differ much from results obtained previously with AGE1.CR cells (the parental cell line for AGE1.CR.pIX) using an AGE1.CR-adapted influenza A/PR/8/34 virus [10].This observation suggests that maximum infectious titers are largely independent of the cultivation mode and that oscillations in the virus titers might also arise in cascades of stirred tank systems [10]. Finally, it is to be expected that in a perfectly mixed tubular bioreactor without diffusion limitations the virus concentration over the tube length should reflect that of a batch culture at different time points post infection. Hence, an ideal tubular bioreactor with perfect mixing and 20 h of nominal RT should show HA and TCID_50_ titers of approximately 2.1 log_10_(HA units/100 μL) and 1×10^9^ TCID_50_/mL at the tube outlet, corresponding to results obtained at 20 h post infection for batch cultivation.

### Production of influenza virus in AGE1.CR.pIX cells in the continuous tubular bioreactor system

#### Cell propagation

**Fig 5:**
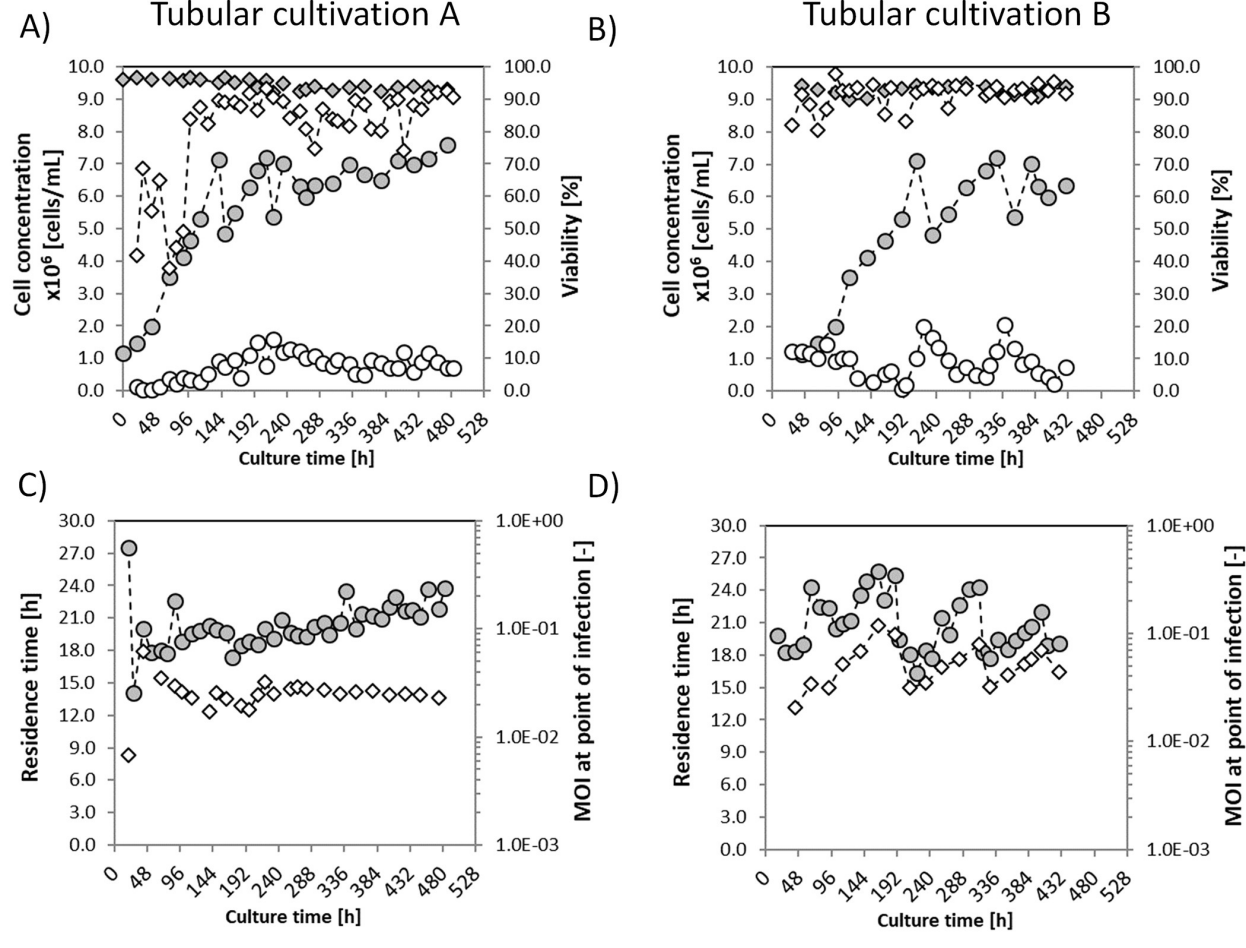
**Cell concentration, viability, residence time and MOI of tubular cultivation A and B for production of influenza A virus in AGE1.CR.pIX.** A) and B), viable cell concentration (circles) and viability (diamonds) of the CSTRs (grey symbols) and the PFBR harvests (open symbols). C) and D) Residence time (circles) of the PFBRs and actual MOI at the POI (diamonds).

Continuous cultivations in the CSTR were started immediately after seeding AGE1.CR.pIX cells at a concentration of 1.0×10^6^ cells/mL, named tubular cultivation A and B (Fig 5A and 5B). Maximum cell concentrations were achieved at 150 h and 200 h for cultivation A and B, respectively. In the PFBR harvest, first cells were visible after 20 h of culture for both cultivations, as expected for the selected RT. A lower cell concentration in the PFBR harvest compared to the CSTR was observed. This can be explained, in part, because of the dilution of F_2_ by F_3_ at the POI (see Fig 1).

Cell viability in the CSTR was always above 90% for both cultivations. In contrast to MDCK.SUS2 cells, no agglomerates were observed for AGE1.CR.pIX cells. In the PFBR harvest, viabilities between 40 and 90% were obtained at the beginning of tubular cultivation A and stabilized between 80–90% after 110 h of culture (Fig 5A). In tubular cultivation B, cell viabilities between 80–90% were measured in the PFBR harvest throughout the experiment (Fig 5B). Lower cell viabilities compared to the CSTR were expected in the PFBR harvest due to the cytopathic effect of an actively propagating virus in the suspension culture inside the tube. However, a less than optimal air supply (F_5_, Fig 1) may explain the low viabilities in the harvest of tubular cultivation A during the first 100 h (Fig 5A), where long liquid segments with lower air-to-liquid ratios were observed.

#### Residence time in the PFBR

With the start of cultivations, the nominal RT of the PFBR was set to 20 h and calculated for each time point (Eq 2). Tubular cultivation A, however, showed a RT of 27 h for the first 24 h of culture, which later stabilized in the range of 20–21 h (Fig 5C). Tubular cultivation B showed an oscillatory pattern of the RT that (after about 20 h) increased to 26 h RT and decreased again to 16 h RT at 225 h of culture (Fig 5D). Afterwards, these oscillations in the RT decreased and approached the nominal RT of 20 h. Visual inspection of the velocity of liquid compartments in the transparent silicone tubes confirmed differences during these RT oscillations. Additional experiments are needed to better understand the origin and impact of these oscillations in process performance. As explained in Materials and Methods, cultivation B was started with a running bioreactor that was flushed with PBS after finishing a previous experiment. The transient stop of pump 1 and 2 for addition of fresh cells in the CSTR and preparation of a fresh VS may have introduced perturbations that significantly affected the pressure inside the already running PFBR. A smooth RT profile like in cultivation A is possible when bioreactor operation is maintained without changes during process operation.

#### MOI in the PFBR

The nominal MOI of the tubular bioreactor system was set to 0.025 as chosen by Frensing et al. [10]. The actual MOI of tubular cultivation A (Fig 5C), was, however, 0.0068 for the first 24 h of culture; later it increased to 0.062 at 50 h of culture, and only then stabilized near 0.025. Obtaining the nominal MOI at the POI was possible, because cells in the CSTR grew to the expected concentration. Thus, no re-adjustment of the infectious titer of the VS was required.

A different situation was obtained in tubular cultivation B, where the actual MOI oscillated between 0.021 and 0.120 during the first 200 h of culture. Afterwards (200–450 h), the MOI was reduced with values between 0.032 and 0.079. This oscillation in the MOI was a result of changes in the cell concentration in the CSTR (Fig 5B at 225 h of culture), which were due to manual corrections (removal of cells from the reactor and dilution with culture medium) in an attempt to maintain the cell concentration near 6×10^6^ cells/mL. Note that the MOI oscillation is not linked to the oscillations in the RT since Eqs 1 and 2 show that MOI and RT are independent parameters. These oscillations in the MOI should be easy to avoid if the cell growth in CSTR can be maintained at steady-state.

#### Virus titers in the PFBR harvest

**Fig 6:**
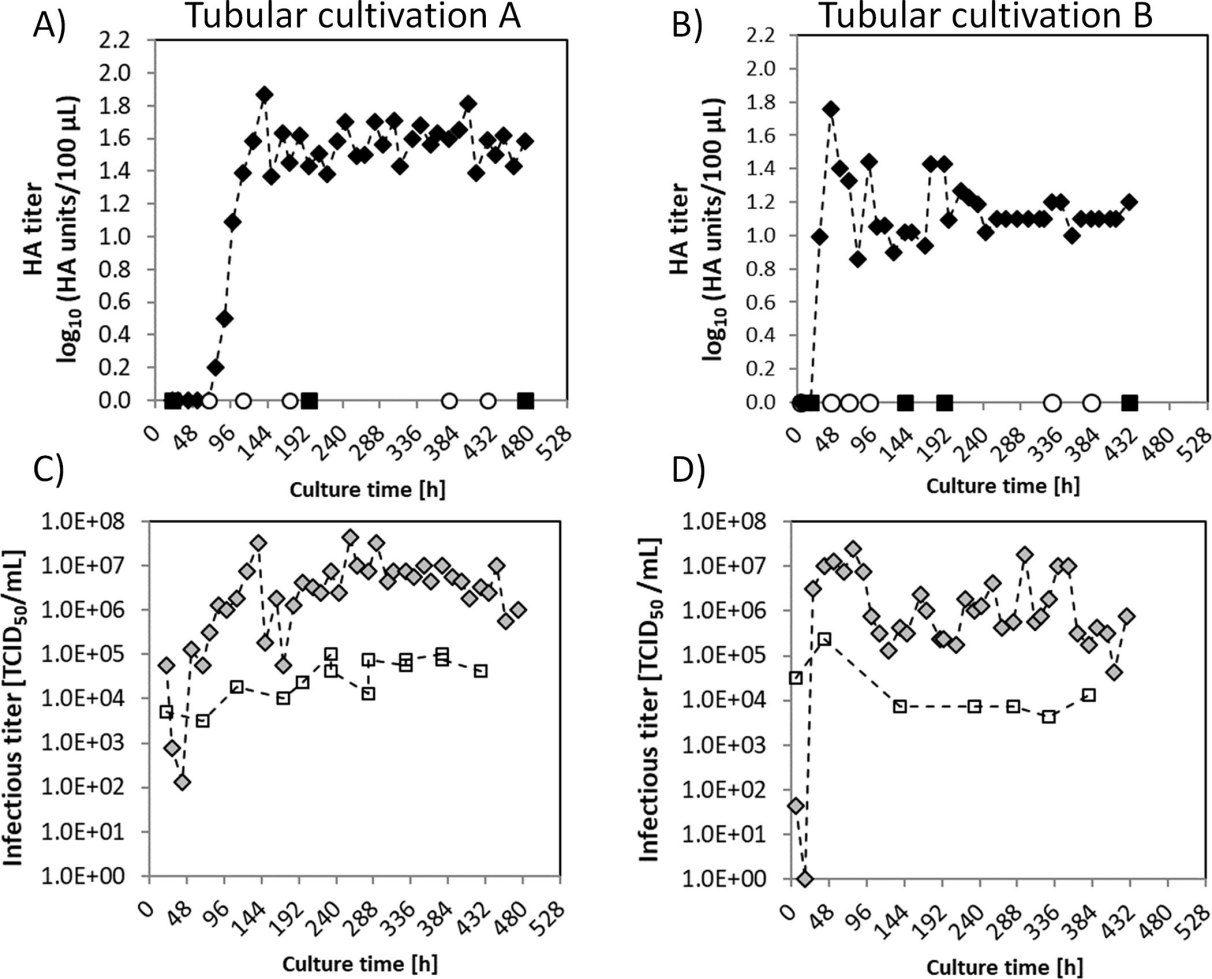
Influenza virus titers in AGE1.CR.pIX cells in the continuous tubular bioreactor system. A) Influenza virus HA titers in the PFBR harvest (closed diamonds), in the VS (open circles) and in the CSTR (closed squares) of tubular cultivation A. B) Influenza HA virus titers in the PFBR harvest, VS and CSTR (same symbols than A) of tubular cultivation B. C) Influenza virus TCID_50_ titers of the harvest (diamonds) and of the VS (squares) of tubular cultivation A. D) Influenza TCID_50_ titers of the harvest and VS of tubular cultivation B (same symbols than C).

HA and TCID_50_ virus titers were measured in the PFBR harvest twice a day (Fig 6A). In tubular cultivation A, HA titers were below the detection limit for the first 60 h and gradually increased after 100 h of culture to values near 1.9 log_10_(HA units/100 μL) to finally stabilize at around 1.6 log_10_(HA units/100 μL) for the rest of the experiment. In the tubular cultivation B (Fig 6B), virus production was observed earlier and an HA value of 1.8 log_10_(HA units/100 μL) was measured at 50 h of culture. Afterwards, HA titers fluctuated between 0.9 and 1.5 log10(HA units/100 μL) and reached a steady-state after 200 h of culture at values near 1.1 log_10_(HA units/ 100 μL). This correlated with a decrease of oscillations in MOI and RT.

Infectious virus titers were measured in the PFBR harvest and in the VS bottle. The VS of tubular cultivation A was maintained at values between 0.9×10^4^ and 1.8×10^5^ TCID_50_/mL during the whole cultivation (Fig 6C). The first infectious titer measured in the PFBR harvest was 1.8×10^4^ TCID_50_/mL and increased to a maximum of 5×10^7^ TCID_50_/mL at 150 h of culture. The infectious titer at the PFBR harvest dropped at 150 h and finally stabilized near a value of 1.0×10^7^ TCID_50_/mL. In tubular cultivation B, the VS was adjusted initially to an infectious titer of 5×10^4^ TCID_50_/mL (Fig 6D) and maintained afterwards at 1×10^4^ TCID_50_/mL. Surprisingly, the TCID_50_ in the PFBR harvest was initially below the limit of quantification (1x10^3^ TCID_50_/mL) for the first 48 h. Then, the infectious titer increased to 1.0×10^7^ TCID_50_/mL to finally stabilize near 1.0×10^6^ TCID_50_/mL.

Overall, the HA titers in the PFBR harvest reached an average value of 1.6 log_10_(HA units/ 100 μL) and 1.1 log_10_(HA units/100 μL) in tubular cultivations A and B, respectively. Compared to virus titer values observed in batch mode (20 h post infection), the HA of the PFBR harvests was clearly reduced (Fig 4B) and corresponded to batch titers expected between 10 and 15 h post infection (Fig 4A). Results indicate that virus propagation in the PFBR was not optimal. Besides virus diffusion limitations, resulting in poor cell-to-cell spreading, other parameters such as the pH inside the tubes and drop in oxygen partial pressure in cell-containing compartments might be relevant. Hence, installing monitoring and controlling ports of dissolved oxygen, pH and metabolite concentration along the PFBR may be useful in future designs, particularly for longer residence times, to ensure oxygen and nutrient supply to the cells. With a Reynolds number in the range 30–60, which corresponds to a laminar regime, and the absence of mechanical mixing, mass transfer is expected to be diffusion limited. Therefore, improving the PFBR harvest titers will require a further optimization of the infection conditions and the tube length. In addition, measures to improve mixing at the POI and along the tube, e.g., passive or static mixing, and incorporation of a vibration or agitation platform should be investigated [27].

The dynamics of the infectious titer in the PFBR harvest was characterized by an initial drop, followed by an increase to values near 1.0×10^7^ and 1.0×10^6^ TCID_50_/mL for tubular cultivation A and B, respectively. At the beginning of cultivations, a similar infectious titer in the harvest and in the VS was measured for up to 60 h. This was an indication of reduced virus replication inside the tube following the bioreactor start-up phase. Afterwards, a TCID_50_ increase of up to three orders of magnitude was achieved compared to the TCID_50_ in the VS.

### Dynamics of replication of influenza virus gene segments 1, 2 and 3

**Fig 7:**
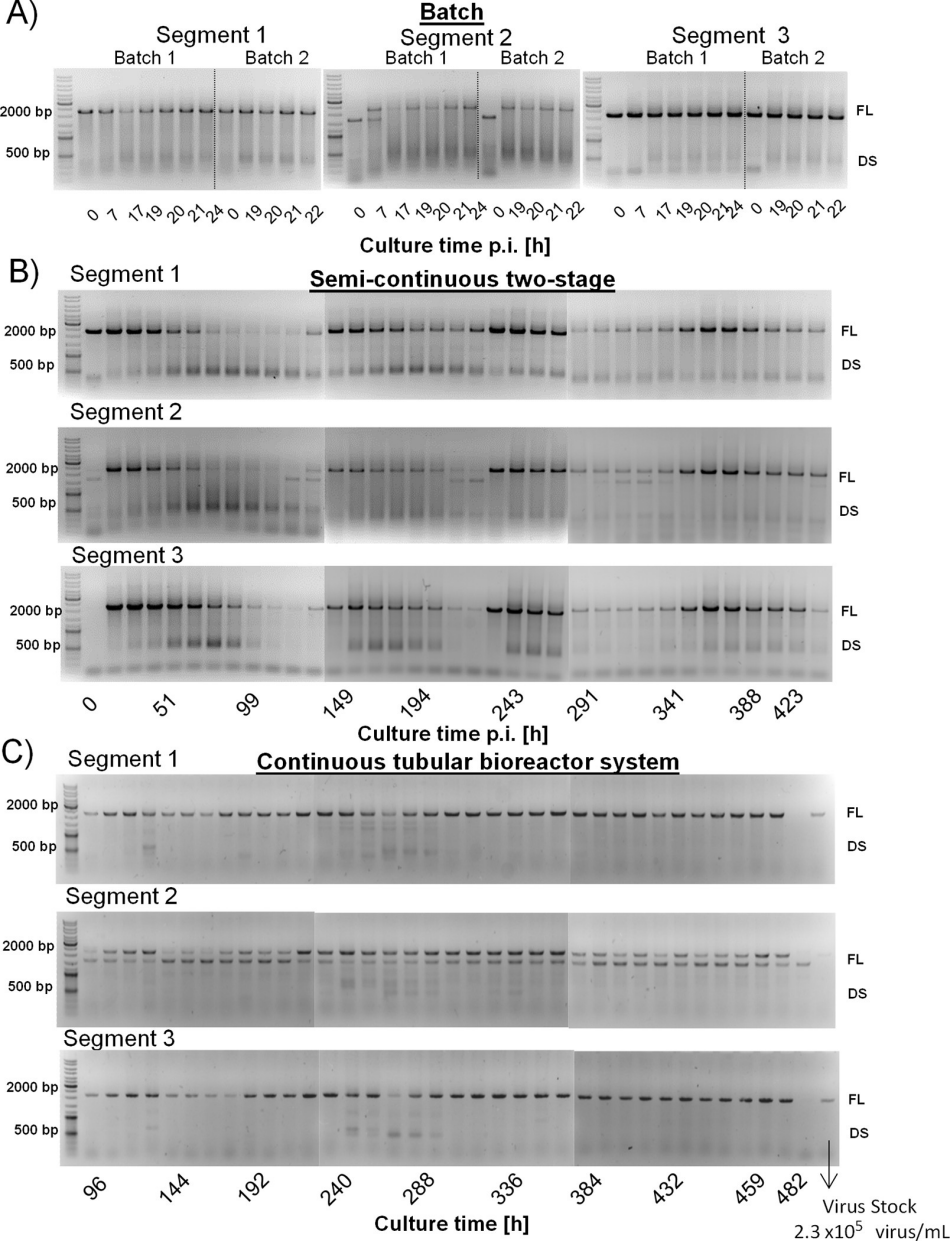
Segment-specific PCR for the detection of full-length and defective genome segments for segment 1, 2 and 3 of influenza A/PR/8/34 (H1N1) virus produced in three different cultivation systems in AGE1.CR.pIX cells. A) Influenza virus propagation in batch mode. B) Influenza virus propagation in a semi continuous two-stage stirred tank system. C) Influenza virus propagation in a continuous tubular bioreactor system with 20 h nominal RT in the PFBR. Each gel left band: reference with ladder 500 bp and 2000 bp 500 bp, each slot corresponds to one time point (sample) of a cultivation. For C) right band: VS used in experiments. FL indicates full-length band for the respective segment, DS defective segments (segments with deletions). The VS was used as a control and included in the PCR of the continuous tubular bioreactor system.

Non-quantitative PCR was used to analyze the dynamics of replication of gene segments 1, 2 and 3 of influenza virus propagated in batch and semi-continuous cultures and the continuous tubular bioreactor system (Fig 7). The PCR assay shows bands of FL segments, and defective segments (segments with deletions, DS) near 2000 bp and 500 bp, respectively.

In batch cultures (Fig 7A), FL segments near 2000 bp were observed for all three genes between 0 and 24 h post infection. Interestingly, a defective segment (less than 2000 bp) was observed at 0 h post infection for segment 2. Later, at 7 h post infection, two bands were observed in the gel, and finally a FL segment with 2000 bp size only (20 h post infection). In general, deletions near 500 bp were generated in all three segments in batch mode, with segment 2 most prone for deletions in the range of 500–2000 bp.

Semi-continuous cultures were maintained for a period of 450 h (Fig 7B). This cultivation mode was selected as a small-scale approach mimicking a true continuous cascade of CSTRs [6]. A more pronounced periodic change in the ratios of FL to respective defective segment was observed when compared to the PCR measurements performed in batch cultures. The PCR signals for all three studied segments appear to oscillate in parallel, most likely due to the interference of FL genome replication by DIPs, and dependence of DIP replication on the presence of the FL segments. A similar replication dynamic was described previously [10] also for cascades of CSTR.

For segment 2, additional copies with a short deletion only (near 2000 bp) were observed at 0, 130, 220, and 320 h post infection that coincides with the results obtained for batch cultures (0 and 7 h post infection, Fig 7A). Whether the parallel oscillations of the various DS segments are due to co-packaging of minor deletions with one dominant genotype, or whether all segments with deletions each can interfere with standard virus replication is not clear yet.

All combined, the control experiments strongly suggest again that a drop in virus titers due to the “von Magnus effect” is a frequent observation in cascades of CSTR for the cell and influenza A virus system analyzed here.

Segment-specific PCR analysis of the virus produced in the continuous tubular bioreactor system (PFBR harvest of tubular cultivation A) is depicted in Fig 7C for a production time of almost 500 h. For this cultivation regime, a surprisingly constant pattern of FL bands was observed for all three segments over the whole duration of the experiment. This pattern differed substantially from the one observed for batch or semi-continuous cultures and suggests the absence of periodic DS dynamics.

Nevertheless, two bands were observed for segment 2 over the whole cultivation period, which was in line with the results observed at early time points post infection in batch culture, as well as for various time points in semi-continuous cultures with infectious titers lower than 1×10^7^ TCID_50_/mL. Assuming that a biological reaction in an ideal well-mixed PFBR follows a batch-like dynamic over the tube length [28], this result indicates that virus spreading and/or intracellular virus replication cycles were not fully completed in the PFBR. Such an effect would suggest that the PFBR is diffusion-limited and, therefore, optimization of MOI and RT (tube length) may be beneficial.

Nevertheless, the continuous tubular bioreactor system provides a virus harvest with a defined virus passage number (in this case, passage number of VS plus one if defined as culture-to-culture transfer) avoiding the accumulation of large numbers of virus segments with deletions, and possible interference with standard virus replication compared to cascades of CSTRs.

### Productivity of the tubular bioreactor system versus batch

The productivity of the tubular bioreactor system can be compared against two hypothetical batch processes, one single-use batch (96 h total production time without cleaning step) and one stainless steel batch process (120 h total production time including 1 day for cleaning and maintenance), in three different scenarios:

#### First scenario: The tubular bioreactor system and the batch processes have the same working volume and are started at the same time

**Fig 8:**
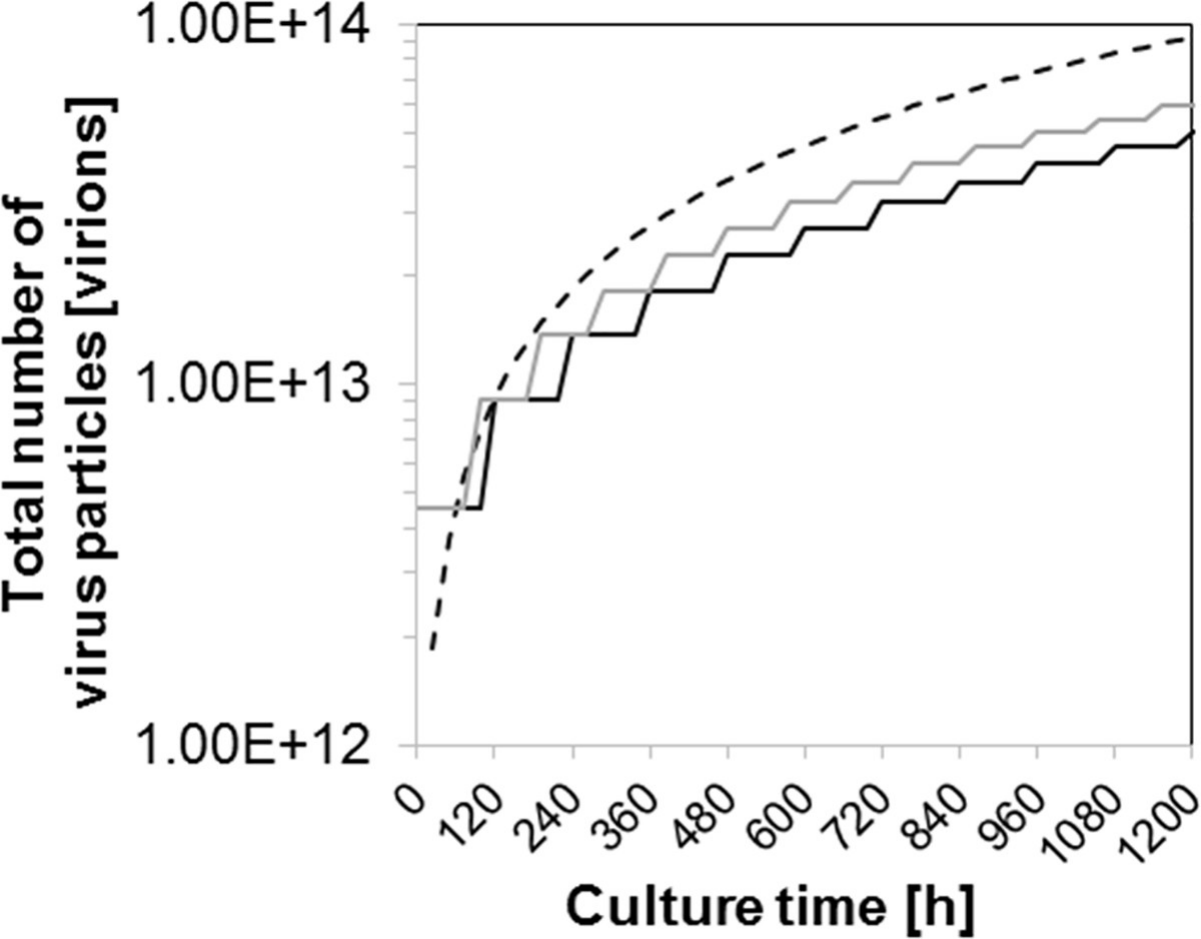
Total number of virus particles produced with a tubular bioreactor system (dashed line) and two batch bioreactors (continuous lines) using suspension MDCK cells. A stainless steel batch (black line) and a single-use batch (grey line) are shown. All cultivation systems with 711 mL working volume, and a maximum virus titer of 6.4×10^9^ virions/mL in the harvest (based on an HA titer of 2.5 log_10_ (HA units/100 μL)). First batch harvest normalized to 0 h; first PFBR harvest at 20 h. Batch cycles of 4 and 5 days for the single-use and stainless steel batch process, respectively.

The total number of influenza virus particles produced (STY multiplied by the accumulated volume and the culture time) is shown in Fig 8. For all cultivation systems a 711 mL WV was considered. For the first batch cycle (normalized to 0 h of culture), both batch processes resulted in a higher number of virus particles than the tubular system. In the second batch cycle, the single-use batch process provided more viruses than the tubular system, and the stainless steel batch provided the same number of virus particles as the tubular system. In the third batch cycle, the tubular system provided a similar number of viruses than the single-use batch process and more viruses than the stainless steel batch bioreactor. This estimation suggests that the batch process can be as efficient as the tubular system for up to two or three batch cycles, depending on whether cleaning and maintenance procedures can be avoided or not. If production exceeds three batch cycles, a tubular system can be more efficient than batch cultures, regardless of the type of batch bioreactors. Note that the concentration of the product in the harvest was assumed to be the same for both production platforms. Hence, the major advantage of the tubular system is the lower time requirement to achieve the same total amount of virus particles.

#### Second scenario: Production of ten times the bioreactor working volume

The production yields of ten times the bioreactor WV with the tubular system and a batch of identical WV (711 mL) was compared. The TY and the STY are shown in Table 1. The TY and the STY of the tubular system were estimated as 7.7×10^10^ virions/h and 1.4×10^10^ virions/(L×h), respectively. The TY and the STY of the single-use batch process were 4.7×10^10^ virions/h and 8.2×10^9^ virions/(L×h), respectively. The TY and the STY of the stainless steel batch process were 3.8×10^10^ virions/h and 6.6×10^9^ virions/(L×h), respectively. As addressed before, while the tubular system needs only 592 h to reach the target volume, the single-use and the stainless steel batch processes require 959 and 1198 h, respectively. Hence, the TY and the STY values of the tubular bioreactor system are approx. two-fold higher.

**Table 1:** Productivity comparison of a continuous tubular bioreactor system versus a batch production system for production of influenza A virus.

Bioreactor type	Description	Bioreactor working volume [mL]	Volumetric production rate [mL/h]^2^	Volume produced [L]	Time required [h]	Maximum cell concentration [cells/mL]	Total cells produced [cells]	Average harvest HA titer log_10_ (HA units/100 μL)^3^	Average harvest virus titer [virions/mL]^4^	Virus production rate [virions/h]	Virus produced [virions]	TY [virions/h]	STY [virions /(L h)]^5^	TY normalized [–]^6^	STY normalized [–]^6^
**Tubular bioreactor system A**^**1**^	Bioreactor developed in this work	711	12	7.1	592	3.0E+06	2.1E+10	2.50	6.4E+09	7.7E+10	4.5E+13	7.7E+10	1.4E+10	2.0	2.1
**Batch A**^**1**^	Stainless steel batch	711	5.93	7.1	1198	3.0E+06	2.1E+10	2.50	6.4E+09	3.8E+10	4.5E+13	3.8E+10	6.6E+09	1.0	1.0
**Batch B**^**1**^	Single-use batch	711	7.41	7.1	959	3.0E+06	2.1E+10	2.50	6.4E+09	4.7E+10	4.5E+13	4.7E+10	8.2E+09	1.3	1.3

Abbreviations: TY = time-yield; STY = space-time-yield; WV = working volume; PFBR = tubular plug flow bioreactor.

^1^ Continuous tubular bioreactor system with a 500 mL CSTR and 211 mL PFBR. Continuous tubular bioreactor system with 3 days of batch cell growth followed by continuous cell propagation. The virus propagation inside the PFBR takes 20 h (1 day); stainless steel batch culture (Batch A) with a cycle of 120 h or 5 days (3 days of cell growth, 1 day of virus propagation, 1 day of cleaning); single-use batch B has a 4 days cycle (no cleaning); the first harvest of both systems is assumed to be taken at 0 of culture (first harvest at outlet of tubular system and first complete batch harvest)

^2^ Production [mL/h] of the continuous tubular bioreactor system as used to design experiments; for batch culture, this value was calculated assuming a production of 711 mL in 4 or 5 days.

^3^ Average haemagglutinin (HA) titer refers to the HA of the total volume of harvest medium collected. For the continuous tubular system the HA value was estimated from HA titers between 336 and 422 h of the experiment with MDCK.SUS2 cells; for batch culture, the HA value was determined from an estimated HA titer at 24 h post infection as described by Lohr et al. 2010 [21].

^4^ Calculated assuming that 1 virus particle binds to 1 cell.

^5^ Liters [L] considers the total volume of medium consumed, including medium required to inoculate the cell bioreactors.

^6^ Normalized to the stainless steel batch culture (Batch A).

#### Third scenario: Scale-up for production of 1000 liters or more

The continuous tubular bioreactor system developed in this work can be scaled-up while keeping three parameters constant: dilution rate in the CSTR, ratio of F_3_/F_2_ (Fig 1), and linear flow velocity in the PFBR (approx. 10 cm/min). Operated at a dilution rate of 0.018 h^-1^ and with a ratio of F_3_/F_2_ = 0.333, a 50 L CSTR would produce cells at a rate of F_2_ = 0.9 L/h (Fig 1) which will be infected with F_3_ = 0.3 L/h. This would result in a total production rate of F_7_ = 1.3 L/h. Regarding the scale-up of the PFBR, the linear velocity inside the tube (volumetric flow rate = velocity × cross-sectional area) can be maintained constant if a 105 m long PFBR with an internal diameter of 2.0–2.5 cm is used. Such 50 L scale tubular bioreactor system would produce 1000 L in 32 days. Alternatively, a tubular bioreactor system built with a 100 L CSTR could produce 1000 L in about 17 days (F_7_ = 2.4 L/h).

## Conclusions

A continuous tubular bioreactor system for production of influenza A virus using the suspension cell lines MDCK.SUS2 and AGE1.CR.pIX was established successfully. The culture format resembles successive bursts of defined single-passage batch processes, where each “batch unit” is compartmentalized by air bubbles. The bioreactor system has a total working volume of 711 mL, requires 1 m^2^ of surface area, and was built using a 105 m long tubular bioreactor. Reynolds numbers between 30 and 60 suggest a laminar regime inside the tube. The bioreactor showed a robust operation over at least three weeks at a nominal flow rate of 0.2 mL/min. As a result, production of 12-times the bioreactor working volume would be possible within one month.

Influenza A/PR/8/34 (RKI) virus was produced successfully with AGE1.CR.pIX cells in two tubular cultivations with 18 days of operation at a nominal MOI of 0.025. Stable HA titers of up to 1.6 log_10_(HA units/100 μL) and infectious titers of up to 1×10^7^ TCID_50_/mL were obtained. PCR analysis of influenza segments 1, 2 and 3 showed that accumulation of defective particles was not significant, and that the “von Magnus effect” can be avoided in such a tubular bioreactor system.

Flow cytometric studies using the suspension cell line MDCK.SUS2 showed that only 16% of the cell population was infected at an MOI of 0.03. At MOI above 0.1 almost all cells were infected and batch-like HA titers were obtained, suggesting that determination of optimal residence time and diffusion within the compartments may be limiting factors. Future designs may improve diffusion by via passive mixing, incorporation of static-mixers or mechanical agitation.

A productivity comparison showed that a continuous tubular bioreactor system can achieve a time-yield and a space-time-yield about two-fold higher than a batch process. Hence, this continuous bioreactor technology can help to increase the volumetric productivity of manufacturing facilities and reduce the footprint of cell culture-based influenza vaccine manufacturing. The evaluation of additional influenza strains in terms of bioreactor infection parameters (infection time, infection cell density, MOI, trypsin amount and temperature), and viral aggregation performance can be considered in future developments. Also, comparing PFBR yields against batch bioreactors across different strains, especially for those flu strains with low production yields, would be an interesting next step to examine. The design presented here prevents the accumulation of defective particles that often accompany the production of an inherently unstable RNA virus. Hence, this continuous and yet single-passage reactor design may also be applicable for production of other cell-based viral vaccines, as well as viral vectors for gene and cancer therapy, provided that the respective viruses can be propagated in suspension cells.

## Abbreviations

CSTR: continuous stirred tank bioreactor
D: dilution rate
DIP: defective interfering particle
DS: defective segment
F: flow rate
FL: full-length
HA: haemagglutinin
MOI: multiplicity of infection [–]
NA: neuraminidase
PFBR: plug-flow bioreactor
POI: point of infection
PBS: phosphate buffer saline
PCR: polymerase chain reaction
Re: Reynolds number [–]
RT: residence time [h]
STV: standard virus
STY: space-time-yield
TCID_50_: 50% tissue culture infectious dose [TCID_50_/mL]
TY: time-yield
VS: virus stock
WV: working volume
